# Intelligent Hierarchical Admission Control for Low-Earth Orbit Satellites Based on Deep Reinforcement Learning

**DOI:** 10.3390/s23208470

**Published:** 2023-10-14

**Authors:** Debin Wei, Chuanqi Guo, Li Yang

**Affiliations:** 1 Communication and Network Laboratory, Dalian University, Dalian 116622, China; weidebin@163.com; 2 School of Automation, Nanjing University of Science and Technology, Nanjing 210094, China

**Keywords:** low-earth orbit, satellite communication, deep reinforcement learning, resource allocation, admission control

## Abstract

Low-Earth orbit (LEO) satellites have limited on-board resources, user terminals are unevenly distributed in the constantly changing coverage area, and the service requirements vary significantly. It is urgent to optimize resource allocation under the constraint of limited satellite spectrum resources and ensure the fairness of service admission control. Therefore, we propose an intelligent hierarchical admission control (IHAC) strategy based on deep reinforcement learning (DRL). This strategy combines the deep deterministic policy gradient (DDPG) and the deep Q network (DQN) intelligent algorithm to construct upper and lower hierarchical resource allocation and admission control frameworks. The upper controller considers the state features of each ground zone and satellite resources from a global perspective, and determines the beam resource allocation ratio of each ground zone. The lower controller formulates the admission control policy based on the decision of the upper controller and the detailed information of the users’ services. At the same time, a designed reward and punishment mechanism is used to optimize the decisions of the upper and lower controllers. The fairness of users’ services admissions in each ground zone is achieved as far as possible while ensuring the reasonable allocation of beam resources among zones. Finally, online decision-making and offline learning were combined, so that the controller could make full use of a large number of historical data to learn and generate intelligent strategies with stronger adaptive ability while interacting with the network environment in real time. A large number of simulation results show that IHAC has better performance in terms of a successful service admission rate, service drop rate, and fair resource allocation. Among them, the number of accepted services increased by 20.36% on average, the packet loss rate decreased by 17.56% on average, and the resource fairness increased by 17.16% on average.

## 1. Introduction

With the continuous advancement of communication technologies and the rapid growth of global communication demands, the existing terrestrial network can provide high-speed and diversified services for densely populated areas, but it still has obvious shortcomings in dealing with long distances, a wide coverage, large capacity, and harsh terrain communication environment. At the same time, as an important supplement to the ground network, a satellite network has significant advantages in expanding the communication coverage, improving the communication capacity and reliability in hot spots, and realizing the space–air–ground–sea integrated network [1,2]. Among them, low-earth orbit (LEO) satellite communication systems have become a project pursued by many enterprises in the Internet, communication, aerospace, and other fields due to its advantages, including a low delay, low cost, high spectrum utilization, and low terminal power requirements [3]. However, LEO satellites are characterized by their low cost, small size, and lightweight design, but they result in severely limited on-board power resources. LEO satellites orbit at low altitudes and move at high speeds, leading to constantly changing coverage areas and complex and dynamic electromagnetic environments. Furthermore, user terminals are unevenly distributed across different countries and regions within the coverage area, and there are varying demands for traditional voice and data services as well as emerging streaming media services. In summary, LEO satellites must address the dynamic changes in user terminal distribution and service demands. Hence, addressing the challenge of allocating resources effectively within the constraints of limited resources such as the satellite spectrum, ensuring equitable admission control for diverse services, and achieving efficient alignment between on-board resources and service demands has emerged as a pressing issue in the realm of satellite communication.

Multi-beam satellite communication technology is an effective approach to solve the above challenges. This technology achieves multiple frequency reuses and polarization reuse through spatial isolation, which can increase satellite communication capacity exponentially. At the same time, the dynamically adjusted beam direction can help the satellite system allocate resources more effectively and further meet the needs of the variability in coverage area and the diversity of communication requirements [4]. However, the flexibility of multi-beam satellite communication systems also increases the complexity of resource allocation. There are usually different communication requirements in the coverage area of each beam, which requires the satellite communication system to be able to sense the differences between beams and dynamically adjust the resource allocation scheme to ensure that the services within each beam are properly served [5]. For these reasons, the academic community has proposed some solutions from the perspectives of beamforming schemes and on-board resource joint allocation.

Lin et al. [6,7] proposed a beamforming scheme for a multi-beam satellite communication system from the perspective of security and energy saving, thereby improving the secrecy energy efficiency (SEE) of communication networks. Lin et al. [8] adopted the alternating optimization scheme and the Taylor expansion penalty function to optimize the beamforming weight vector and phase shift, aiming to minimize the total transmission power of the satellite and the base station, while meeting the user rate requirements. Deng et al. [9] proposed an adaptive packet splitting scheme based on a discrete firefly algorithm for cross-layer and cross-dimension radio resources optimization and an irregular gradient algorithm to ensure communication efficiency and reliability. Zhang et al. [10] proposed an uplink cooperative user scheduling and power allocation method based on game theory in an uplink multi-beam satellite Internet of Things (S-IoT). Takahashi et al. [11] constructed a power resource allocation model to jointly control the transmit power and multi-beam directivity according to the traffic demand, so as to improve the efficiency of communication resource allocation. Jia et al. [12] considered the inter-beam interference, channel conditions, delay, capacity, bandwidth utilization variance, and other factors, and a joint resource allocation algorithm is proposed, which can flexibly allocate resources according to specific service requirements and channel conditions. Wang et al. [13] proposed a resource management scheme to maximize resource utilization and user service weight, and introduced an improved cuckoo optimization algorithm to ensure the quality of service (QoS) for high-priority users. In addition, Cai et al. [14] proposed a cognitive S-IoT system supporting code division multiple access (CDMA), which can jointly optimize the transmission power of the traditional satellite system and the IoT by maximizing the total rate of the IoT users under the premise of ensuring the performance requirements of the traditional satellite system.

The emergence of the above research results brings new ideas and methods for the resource allocation of multi-beam LEO satellite communication. However, when dealing with the diverse and time-varying communication requirements in the network, these methods are difficult for meeting the needs of rapid and dynamic resource allocation due to high algorithm complexity and long calculation times. Machine learning (ML) [15] has super strong learning and inference capabilities, and has been shown as a good application prospect in network resource allocation [16,17,18,19]. In particular, the introduction of deep reinforcement learning (DRL) provides a more intelligent, flexible, and efficient solution for the resource allocation of multi-beam LEO satellite communication.

Liao et al. [20] designed a cooperative multi-agent deep reinforcement learning (CMDRL) framework to solve the bandwidth allocation problem of multi-beam satellite communication systems. Chan et al. [21] proposed an efficient power and bandwidth allocation method, which uses two linear ML algorithms and takes channel conditions and traffic demand as inputs to achieve the optimal resource allocation under dynamic channel conditions. Hu et al. [22] considered inter-beam interference and resource utilization difference, and a game theory based on bandwidth allocation model for forward links was established. Furthermore, considering that the addition of satellite beams results in a larger action space for individual agents in DRL, thereby increasing time complexity, this paper proposed a multi-agent cooperative deep reinforcement learning approach to achieve optimal bandwidth allocation. He et al. [23] proposed a multi-objective deep-reinforcement-learning-based time-frequency (MODRL-TF) two-dimensional resource allocation algorithm to achieve the joint optimization goal of maximizing the number of users and system throughput for the joint allocation problem of multi-dimensional resources such as QoS, time, and frequency in multi-beam satellite communication. Huang et al. [24] proposed a learning-based hybrid-action deep Q-network (HADQN) algorithm to solve the sequential decision optimization problem in dynamic resource allocation in multi-beam satellite systems. By using a parameterized hybrid action space, HADQN can schedule the beam pattern and allocate the transmitter power more flexibly, which greatly reduces the on-orbit energy consumption without affecting the QoS.

By introducing DRL, the above research provided a forward-looking solution for solving resource allocation in multi-beam LEO satellite networks. However, current research still has some shortcomings, with the main deficiency being the lack of consideration for fair user service admission in the optimal allocation of resources. In the multi-beam LEO satellite communication system, the coverage area of the satellite is constantly changing, and the needs of users in the area are diverse.

If fairness is not considered in resource allocation, this will result in some users being overlooked or frequently denied access, while others consistently occupy more resources. Over time, this leads to a significant decline in user experience, causing dissatisfaction with the services provided by the satellite. However, in the existing research, traditional algorithms exhibit limited flexibility and adaptability when confronted with the complexities of the satellite communication environment. Moreover, accurately extracting the key information required for fair admission control strategies from a vast array of service demand characteristics often proves challenging. Additionally, a comprehensive and effective hierarchical admission control strategy capable of achieving the cooperative optimization of resource allocation rationality and admission control fairness has yet to be established. Hence, we propose an intelligent hierarchical admission control (IHAC) strategy based on DRL. This approach incorporates the user service model, the dynamic priority model, and carefully designed controller feature inputs, along with a reward and punishment mechanism. Through the utilization of DRL, IHAC aims to efficiently allocate multi-beam resources and exercise control over user service access within the coverage area, simultaneously addressing both objectives.

The contributions of this paper are as follows:

- In this paper, the DLR method is employed to effectively manage the multi-beam satellite communication system. By combining deep deterministic policy gradient (DDPG) and deep Q-network (DQN) algorithms, a hierarchical admission control strategy is constructed, encompassing both upper- and lower-level controllers. The upper-level controller employs DDPG to comprehensively consider the service demands of various ground zones and formulate resource scheduling strategies from a global perspective. This ensures optimized resource utilization while ensuring the coordinated allocation of beam resources among different ground zones. The lower-level controller employs DQN to receive decisions from the upper-level controller and refines the global strategy into localized admission control strategies for each ground zone’s services.
- This paper develops user service models and dynamic priority models for services. The user service model enables controllers to have a deeper understanding of different types of service characteristics, leading to more precise decision-making. By introducing the adaptive dynamic priority model, controllers can achieve efficient resource utilization while considering the fairness of competitive services, leading to a more balanced resource allocation. Furthermore, careful design is applied to the feature inputs, reward functions, and penalty terms of both upper- and lower-level controllers, facilitating a more comprehensive and detailed understanding of service demands. This guides controller decisions towards optimizing both efficient resource utilization and overall fairness of the communication system.
- In simulation experiments, the proposed strategy is compared with DAQC [25] and WOA [26]. The results demonstrate that the proposed strategy performs well in terms of the channel access service quantity, successful service admission rate, service drop rate, and resource allocation fairness. This highlights the effectiveness of the proposed strategy.

The remainder of this paper is organized as follows. Section 1 describes the related works on resource allocation and admission control for multi-beam satellites and the motivation of this paper. Section 2 describes the system model of the resource allocation and admission control strategy in this paper. Section 3 outlines the hierarchical admission control framework based on DRL. Section 4 provides a detailed explanation of the hierarchical admission control strategy. Section 5 presents the evaluation results of the experiments. Section 6 summarizes the entire paper and elaborates on the contributions of this work.

## 2. System Model

### 2.1. Communication Model of the Satellite Coverage Area System

In LEO satellite communication systems, as the satellite moves in the orbit, its coverage will change, and the corresponding traffic load and service priorities will also change. In order to meet the changing requirements of ground tasks, it is necessary to allocate satellite resources reasonably. The resource allocation problem is mainly affected by time window constraints, satellite power consumption constraints, ground user priorities, etc.

**Figure 1:**
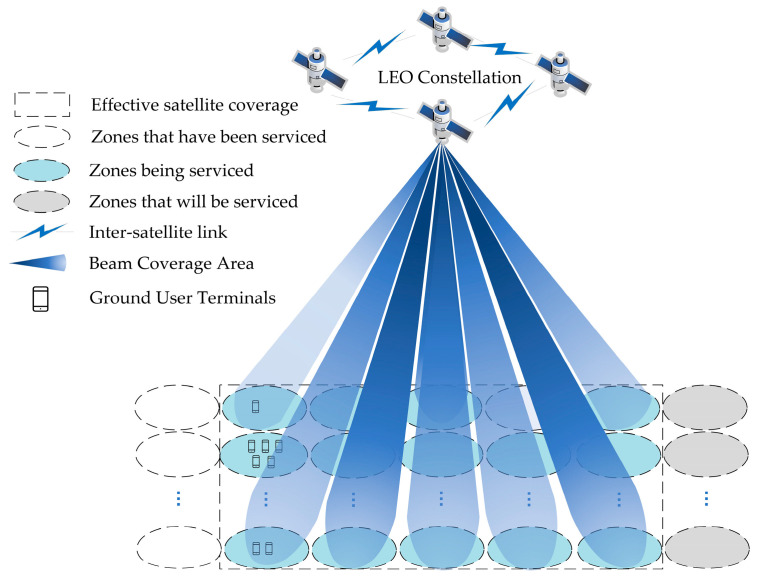
Multi-beam satellite communication system model.

The system model for multibeam LEO satellite communication is illustrated in Figure 1. We simplify the satellite coverage area into rectangles and further divide it into *M* fixed ground zones, and the communication tasks are assigned to different ground zones according to the location of their initiating users. In the context of multibeam satellite communication, LEO satellites allocate their transmission capacity into multiple beams, each directed towards a specific ground zone. LEO satellites can dynamically adjust the beam resources of ground zones in real-time based on the distribution of services on the ground, and further decide whether to admit user services within these zones. This communication architecture between user terminals and satellites forms a complex system.

### 2.2. User Service Model

The core focus of this paper is to address the complex issue of user service requests within multibeam satellite communication systems. The initial step involves constructing a user service model to depict various types of user services. This user service model comprehensively accounts for various parameters and features of the requested services to ensure a comprehensive and accurate representation of user service requirements. In addition to the user model, we also establish a dynamic user service priority model. Given the constantly changing communication environment, service priorities are adjusted based on dynamically changing conditions, such as the remaining service value and service urgency. By introducing an adaptive dynamic priority mechanism, the user service admission process is further optimized to ensure efficient resource utilization, while achieving fair resource allocation among competitive services. Among them, the relevant variable descriptions of user services are presented in Table 1.

With the movement of satellites and the progression of time, the status of user services is constantly changing. We design a sextuple to represent the data model of user services within a beam. Let there be M ground zones within the satellite coverage rectangular area, each containing N user services. To account for the service status of the satellite beam, the user services $U_{m,n}$ within the beam coverage area are as follows.

(1) $$U_{m,n}=(B_{m,n},P_{m,n},C_{m,n},V_{m,n},Ts_{m,n},S_{m,n}).$$

where m∈{1,2,…,M}, n∈{1,2,…,N}; $B_{m,n}$ and $P_{m,n}$ satisfy the condition $\sum_{m=1}^{M}\sum_{m=1}^{N}B_{m,n}\leq B_{total}$, $\sum_{m=1}^{M}\sum_{n=1}^{N}P_{m,n}\leq P_{total}$; the initial priority $V_{m,n}$ is from the set {1,2,3,4,5}, corresponding to background flow service, interactive service, streaming media service, audio service, and handover service; $S_{m,n}$ satisfies the condition $ST\leq Ts_{m,n}\leq ET$, $S_{m,n}\leq ET-ST$.

Building upon the initial priority of user services, this paper also takes into account the aspects of the remaining service value and the urgency of service provisioning. The dynamic priority $DP_{m,n}$ is defined as:

(2) $$DP_{m,n}=V_{m,n}\times \frac{t-t^{\prime }}{ET-t+1}$$

Let $t_{start}$ be the time that the service be served. If the service has been served, then $t^{\prime }=t_{start}$; if the service has not been served, then $t^{\prime }=Ts_{m,n}$, and $S_{m,n}\leq ET-t$. According to Equation (2), the dynamic priority of the user service is directly proportional to the initial priority of the service and the time for the service to be served, and inversely proportional to the remaining time of this resource allocation time window. The dynamic priority introduced in this paper enables the strategy to comprehensively understand and evaluate service priorities, leading to more intelligent service management and admission decisions.

## 3. Framework of Intelligent Hierarchical Admission Control Based on DRL

### 3.1. Overview

DRL, as a powerful artificial intelligence technique, offers the core advantage of autonomous learning and optimization in complex and uncertain environments. It achieves this by interacting with the environment, accumulating experiences, and progressively improving decision quality [27,28,29,30].

The integration of the DDPG algorithm and DQN algorithm, which are prominent components of DRL, provides a novel solution for hierarchical admission control in satellite communication systems. DDPG extends the concepts of DQN and the Actor-Critic framework, effectively handling the complexity of state spaces, making it particularly suitable for the continuous action control problem of allocating beam resources to ground zones. On the other hand, DQN can accurately perceive and analyze the state features and communication demands of user services, addressing the discrete admission control problem. Combining these DRL algorithms with LEO satellite communication hierarchical admission control leads to intelligent decisions regarding user admission into the communication network and optimal resource allocation under limited resource conditions.

**Figure 2:**
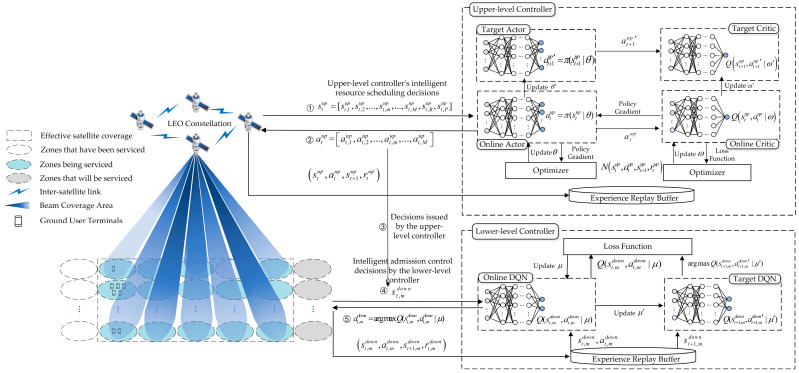
Intelligent hierarchical admission control framework.

The combination of the above DRL algorithms, LEO satellite communication resource allocation, and admission control forms a two-level intelligent admission control framework, as illustrated in Figure 2. In this framework, the upper-level controller bears the responsibility of intelligent decision-making on beam resource allocation for ground zones based on their status features and communication demands. Meanwhile, the lower-level controller focuses on the admission control of user services within each zone. Guided by upper-level decisions and considering the actual situations of user services in each zone, the lower-level controller manages communication resources more delicately, ensuring a certain level of fairness in the allocation of user service resources. Among them, the parameters in the DRL modeling process are defined in Table 2.

Within the context of this hierarchical admission control strategy, it is necessary to holistically consider the relationships among all ground zones, while also giving due consideration to the priority and fairness management among user services. The implementation of this strategy introduces a new level of intelligence to the entire communication system, allowing the system to better adapt to diverse communication needs and provide users with a higher-quality communication experience.

### 3.2. DRL Formulation

#### 3.2.1. State

As illustrated in Figure 2, the upper-level and lower-level controllers are respectively equipped with DDPG and DQN neural networks, with DDPG further divided into Actor and Critic neural networks. The neural networks are required to make decisions based on the state features of satellite communication. To enhance the neural networks’ perception capabilities concerning ground zones, user service states, and communication demands, ensuring that the admission control strategy maintains fairness across various service resource allocations, we have specially designed the input features for the upper- and lower-level neural networks. These input features are crafted to provide richer information, enabling the neural networks to make more precise decisions across diverse scenarios, thereby effectively optimizing the performance and resource allocation of the satellite communication system.

In the upper-level admission control strategy, each ground zone initially conducts unified scheduling and management of its internal services. After analyzing and processing user service information, the ground zone’s state features are formed and submitted to the upper-level DDPG. The input features encompass both user service and ground zone information, such as the number of service requests in the ground zone, total dynamic priority, and aggregate requested bandwidth. This empowers the DDPG to gain comprehensive insights into interactions and communication needs among users within the ground zone, thus providing more precise guidance for higher-level decisions. Meanwhile, the input features for the lower-level DQN include the upper-level DDPG’s resource allocation strategy for the ground zone and more detailed user service information, such as service types. This design enables the lower-level DQN to more accurately assess each user’s communication performance, enabling more targeted admission control decisions.

To be specific, the upper-level DDPG’s input features are denoted as $s_{t}^{up}=(s_{t,1}^{up},s_{t,2}^{up},\ldots ,s_{t,m}^{up},\ldots ,s_{t,M}^{up},s_{t,B}^{up},s_{t,P}^{up})$, where $s_{t,m}^{up}$ signifies the state features of the *m*th ground zone, and $s_{t,B}^{up}$ and $s_{t,P}^{up}$ represent the satellite’s total bandwidth and total power, respectively. The lower-level DQN’s input features are represented as $s_{t,m}^{down}$.

(3) $$s_{t,m}^{up}=(N_{m},Pr_{m},Wt_{m},B_{m},P_{m},C_{m}).$$

(4) $$s_{t,m}^{down}=({\hat{B}}_{m,n},{\hat{P}}_{m,n},{\hat{C}}_{m,n},V_{m,n},Ts_{m,n},S_{m,n},DP_{m,n},a_{t,m}^{up}).$$

The specific features are given by the following formulas.

(5) $$Pr_{m}=\sum_{n=1}^{N_{m}}DP_{m,n}.$$

(6) $$Wt_{m}=\sum_{n=1}^{N_{m}}\left(t-Ts_{m,n}\right).$$

(7) $$B_{m}=\sum_{n=1}^{N_{m}}B_{m,n}.$$

(8) $$P_{m}=\sum_{n=1}^{N_{m}}P_{m,n}.$$

(9) $$C_{m}=\sum_{n=1}^{N_{m}}B_{m,n}\;\log_{2}(1+SINR_{m}).$$

$SINR_{m}$ represents the signal-to-interference-plus-noise Rrtio (SINR) of the *m*th beam:

(10) $$SINR_{m}=\frac{A_{m}\sum_{n=1}^{N_{m}}P_{m,n}}{N_{0}\sum_{n=1}^{N_{m}}B_{m,n}+\sum_{i\in M,i\neq m}A_{i}\sum_{n=1}^{N_{i}}P_{i,n}}.$$

where $A_{m}$ is the channel attenuation coefficient of the *m*th ground zone; $N_{0}$ is the power spectral density of additive white Gaussian noise; $\sum_{i\in M,i\neq m}A_{i}\sum_{n=1}^{N_{i}}P_{i,n}$ is the sum of co-channel interference from other beams to the mth beam.

The ratios of the requested bandwidth, power, and capacity of the nth user service to the total requested resources in the mth ground zone ${\hat{B}}_{m,n}$, ${\hat{P}}_{m,n}$, and ${\hat{C}}_{m,n}$ are as follows:

(11) $$\begin{array}{l} {\hat{B}}_{m,n}=B_{m,n}/B_{m},\\ {\hat{P}}_{m,n}=P_{m,n}/P_{m},\\ {\hat{C}}_{m,n}=C_{m,n}/C_{m}. \end{array}$$

#### 3.2.2. Action

In the proposed hierarchical admission control architecture, the upper- and lower-level controllers are responsible for the scheduling of satellite-to-ground beam resources and the admission control of user services within ground zones, respectively. The upper-level controller plays a crucial role in global decision-making. It aggregates network state features from various ground zones and feeds this information into the DDPG neural network. Subsequently, based on these network state features, DDPG formulates a set of resource scheduling policies $a_{t}^{up}=(a_{t,1}^{up},a_{t,2}^{up},\ldots ,a_{t,m}^{up},\ldots ,a_{t,M}^{up})$ for each ground zone, where $a_{t,m}^{up}\in [0,1)$, $\sum_{m=1}^{M}a_{t,m}^{up}=1$, and $a_{t,m}^{up}$ represent the scheduling policy for the mth ground zone’s beam resources.

Specifically, when $a_{t,m}^{up}\in (0,1)$, it indicates that the satellite decides to admit the mth ground zone and allocate satellite resources to the beams of the current ground zone according to a certain percentage. On the other hand, when $a_{t,m}^{up}=0$, it signifies that the satellite will not admit that ground zone.

Once the satellite completes the resource scheduling for various ground zones, it enters the domain of the lower-level controller. The primary responsibility of the lower-level controller is the admission control of user services within each ground zone. At this stage, the lower-level DQN leverages the state feature $s_{t,m}^{down}$ of user services within its respective ground zone to formulate admission control policies $a_{t,m}^{down}\in \{0,1\}$ for each user service. When $a_{t,m}^{down}=1$, it signifies that the corresponding user service is admitted, whereas $a_{t,m}^{down}=0$ indicates that the user service is not admitted.

#### 3.2.3. Reward

In the proposed intelligent hierarchical admission control framework based on DRL, the reward function plays a crucial role. The design of this key element not only establishes a close correlation between the network state feature input and the admission control policy output but also guides the neural network towards rapidly approaching and converging to the predefined optimization objectives. During the resource scheduling process for various ground zones by the satellite, the design of the reward function for the upper-level DDPG needs to take into account the overall system performance, ensuring both communication system efficiency and fairness. This prevents excessive bias of communication resources towards specific ground zones, which could impact the overall communication effectiveness. Therefore, the reward function $r_{t}^{up}$ for the upper-level DDPG is formulated as follows:

(12) $$r_{t}^{up}=\alpha_{1}\sqrt{\frac{\sum_{m=1}^{M}{\left(SPR_{m}-SPR_{ave}\right)}^{2}}{M}}+\alpha_{2}\sqrt{\frac{\sum_{m=1}^{M}{\left(DWT_{m}-DWT_{ave}\right)}^{2}}{M}}.$$

where $\alpha_{1},\;\alpha_{2}$ are weight coefficients that weigh the importance of two terms in (12). The reward function $r_{t}^{up}$ comprehensively evaluates the fairness of the satellite’s admission control strategy $a_{t}^{up}$ for various ground zones by considering the variance of the total dynamic priority of successfully admitted services $\sqrt{\frac{1}{M}\sum_{m=1}^{M}{\left(SPR_{m}-SPR_{ave}\right)}^{2}}$ and the variance of the total waiting time of services not admitted across all ground zones $\sqrt{\frac{1}{M}\sum_{m=1}^{M}{\left(DWT_{m}-DWT_{ave}\right)}^{2}}$.

The lower-level DQN is built upon the foundation of the upper-level admission control strategy and aims to make decisions for services within each ground zone. In this architecture, a reasonable lower-level admission control strategy’s primary concern is to ensure compliance with the resource allocation proportions set by the upper level. If the total resource consumption of admitted services exceeds these limits, the lower-level DQN reward function needs to impose a certain penalty to maintain system balance and stability. Similarly, the fairness of the intra-zone service admission control strategy must also be ensured. Therefore, we employ the total dynamic priority $SPR_{m}$, the total waiting time of successfully admitted services $SWT_{m}$, and the reward or penalty $RP_{m}$ for the resource utilization rate of the ground area admission control strategy to design the reward function $r_{t}^{up}$ for the lower-level DQN:

(13) $$r_{t,m}^{down}=\beta_{1}SPR_{m}+\beta_{2}SWT_{m}+\beta_{3}RP_{m}$$

To ensure data consistency, we have normalized these values. Through these two components, the lower-level DQN is guided to balance the dynamic priority and waiting time of intra-zone user services. Additionally, $RP_{m}$ is obtained as follows:

(14) $$RP_{m}=\left\{\begin{array}{l} -500,RU_{m}=0\\ 10\log_{1.1}(RU_{m}),0<RU_{m}<1\\ 10,RU_{m}=1\\ 10\log_{0.9}(RU_{m}),RU_{m}>1 \end{array}\right..$$

where $RU_{m}$ represents the ratio of resources allocated to user services within the mth ground zone to the resources allocated by the upper-level control strategy.

## 4. Strategy of Intelligent Hierarchical Admission Control Based on DRL

### 4.1. Hierarchical Controller Structure

We delve into the exploration of DDPG and DQN, evolved from the traditional Q-learning algorithm, and their collaborative implementation in the hierarchical admission control framework. However, these methods also inherit issues such as instability, overestimation, and convergence challenges that have been associated with traditional Q-learning, especially when utilizing neural networks to estimate Q-values.

In conventional Q-learning, a common practice involves using a single online network to estimate Q-values for each state-action pair, and decisions are made based on the action with the highest Q-value. Nevertheless, this approach can lead to unstable Q-value estimations, particularly during the initial stages of training. This instability arises from the chain reaction triggered by the parameter updates in the neural network, resulting in fluctuations in Q-values. Furthermore, as traditional Q-learning employs a greedy strategy to select actions with the highest Q-values, it can lead to the overestimation of Q-values, subsequently affecting the accuracy of the policy.

To overcome these challenges, we introduce the concepts of a target network and experience replay mechanism in DRL. Specifically, the target network shares the same structure as the online network but employs a different parameter update strategy. The target network’s parameters are copied from the online network at regular intervals or through a soft update approach. This separation of neural networks ensures that the calculation of the target network Q-values is unaffected by the direct influence of the online network, reducing the immediate impact of the online network’s instability and mitigating the chain reaction caused by parameter updates.

Furthermore, in interactive environments, the acquired data are often highly correlated, with potential correlations between consecutive data points. This correlation can lead to the neural network being influenced by related data during the training process, thereby causing instability. The experience replay mechanism, by randomly sampling from an experience buffer, breaks down data correlation, enhancing data utilization efficiency. The deployment of neural networks is as follows.

#### 4.1.1. DDPG in the Upper-Level Controller

Online Actor Network: $\pi (s_{t}^{up}|\theta )$, where θ represents the neural network parameters. Responsible for generating the scheduling strategy $a_{t}^{up}$ for various ground zones’ beam resources based on the ground zone and satellite resource state features $s_{t}^{up}$.

Online Critic Network: $Q(s_{t}^{up},a_{t}^{up}|\omega )$, where ω represents the neural network parameters. Primarily evaluates the quality of actions $a_{t}^{up}$ made by the online actor network based on $s_{t}^{up}$, guiding the optimization of the action network’s decision-making capability.

Target Actor Network and Target Critic Network: $\pi (s_{t+1}^{up}|\theta^{\prime })$ and $Q(s_{t+1}^{up},a_{t+1}^{up}{}^{\prime }|\omega^{\prime })$, where $\theta^{\prime }$ and $\omega^{\prime }$ are the parameters of these two neural networks, and $a_{t+1}^{up}{}^{\prime }$ is the action computed by the target action network based on $s_{t+1}^{up}$, but not executed during the interaction process.

#### 4.1.2. DQN in the Lower-Level Controller

Online DQN: $Q(s_{t,m}^{down},a_{t,m}^{down}|\mu )$, where μ represents the neural network parameters. In the distributed scenario of ground zone user service admission control, each ground zone possesses an independent online DQN, sequentially making admission control decisions for the user service within its zone.

### 4.2. DRL Training

While interacting with the environment, the upper- and lower-level controllers accumulate a variety of state-action pairs’ experience samples $\left(s_{t}^{up},a_{t}^{up},s_{t+1}^{up},r_{t}^{up}\right)$ and $\left(s_{t,m}^{down},a_{t,m}^{down},s_{t+1,m}^{down},r_{t,m}^{down}\right)$ by trying different actions. These experience samples encompass the behavior and outcomes of the satellite communication system under various states. They are stored in an experience replay pool for neural network training. By obtaining $N(s_{i}^{up},a_{i}^{up},s_{i+1}^{up},r_{i}^{up})$ and $N(s_{i,m}^{down},a_{i,m}^{down},s_{i+1,m}^{down},r_{i,m}^{down})$ through random sampling, the correlation between samples is broken, thereby enhancing training stability and the system’s understanding and adaptability to different situations.

#### 4.2.1. Training of the Upper-Level Controller DDPG

During the training phase of DDPG, the online actor network utilizes the experience samples $N(s_{i}^{up},a_{i}^{up},s_{i+1}^{up},r_{i}^{up})$ from the replay pool to update its weights based on Equation (15):

(15) $$\nabla_{\theta }J\approx \frac{1}{N}\sum_{i=1}^{N}\nabla_{a}Q(s_{i}^{up},a_{i}^{up}|\omega )\nabla_{\theta }\pi (s_{i}^{up}|\theta ).$$

The online actor network relies on the backpropagation of the policy gradient $\nabla_{\theta }J$ for updates. Additionally, the online critic network can be trained by minimizing the loss function, as expressed below:

(16) $$Loss(\omega )=\frac{1}{N}\sum_{i}^{N}{\left(y_{i}^{up}-Q(s_{i}^{up},a_{i}^{up}|\omega )\right)}^{2},$$

where $y_{{}_{i}}^{up}$ is referred to as the target Q-value, which can be expressed in a sample $\left(s_{i}^{up},a_{i}^{up},s_{i+1}^{up},r_{i}^{up}\right)$ as follows:

(17) $$y_{{}_{i}}^{up}=r_{i}^{up}+\gamma Q(s_{i+1}^{up},a_{i+1}^{up}{}^{\prime }|\omega^{\prime }),$$

The target Q-value is obtained by taking a weighted sum of the current reward $r_{i}^{up}$ and $Q(s_{i+1}^{up},a_{i+1}^{up}{}^{\prime }|w^{\prime })$, where γ is considered the discount factor, and $a_{i+1}^{up}{}^{\prime }$ is computed by the target action network, expressed as follows:

(18) $$a_{i+1}^{up}{}^{\prime }=\pi (s_{i+1}^{up}|\theta^{\prime }).$$

Finally, we adopted a soft update approach for updating the parameters of both target networks, where in each update iteration, the parameters are updated by weighting them with the learning rate τ, expressed as follows:

(19) $$\begin{array}{l} \omega^{\prime }=\tau \omega +(1-\tau )\omega^{\prime },\\ \theta^{\prime }=\tau \theta +(1-\tau )\theta^{\prime }. \end{array}$$

#### 4.2.2. Training of the Lower-Level Controller DQN

Given that we have adopted a distributed ground zone approach with multiple intelligent agents and treated each ground zone as an individual agent, in this architecture, each ground zone needs to balance resource allocation and services admission decisions while collaborating and interacting with others. This involves finding a balance between cooperation and competition among multiple intelligent agents. Moreover, we recognize that the upper-level controller comprehensively considers the dynamic situations of each ground zone based on global information, thereby partially balancing the resource competition process among them and indirectly providing a global perspective for the lower-level controller. Therefore, we opt for a decentralized training approach for the deployment of lower-level controller agents. In this scheme, each ground zone’s agent is relatively independent in its training, making decisions based on local information. Here, we provide an overview of the training process for a single intelligent agent.

The lower-level controller primarily focuses on training the online DQN, and its principle is similar to training the online critic network in the upper-level controller. Similarly, based on the sample $N(s_{i,m}^{down},a_{i,m}^{down},s_{i+1,m}^{down},r_{i,m}^{down})$, we train the online DQN by minimizing the loss function:

(20) $$Loss(\mu )=\frac{1}{N}\sum_{i}^{N}{\left(y_{i,m}^{dowm}-Q(s_{i,m}^{down},a_{i,m}^{down}|\mu )\right)}^{2},$$

(21) $$y_{i,m}^{dowm}=r_{i,m}^{down}+\gamma Q(s_{i+1,m}^{down},a_{i+1,m}^{down}{}^{\prime }|\mu^{\prime }),$$

(22) $$a_{i+1,m}^{down}{}^{\prime }=\pi (s_{i+1,m}^{down}|\mu^{\prime }).$$

The parameter update of the target DQN still adopts the soft update method:

(23) $$\mu^{\prime }=\tau \mu +(1-\tau )\mu^{\prime }.$$

### 4.3. Intelligent Hierarchical Admission Control Strategy

Considering the characteristics of satellite high-speed movement and the real-time variability of users’ needs and demands, we have adopted a strategy of online decision-making and offline learning for the training and decision-making of intelligent agents. This strategy aims to ensure that the agents can make adaptive decisions in real-time environments and continuously enhance their decision-making capabilities through offline learning.

During the online decision-making phase, the intelligent agents interact with the environment through the upper and lower-level controllers, accumulating experiences. This real-time interaction process enables the agents to promptly perceive changes in the satellite movement, the distribution of user services, and the actual utilization of resources. In the offline learning phase, we utilize these accumulated experiences to optimize the agents’ decision-making capabilities. By deeply learning and analyzing the experiential data, the agents can acquire insights into more intricate patterns and trends. The detailed strategy process is illustrated in Table 3.

Overall, the dual approach of online decision-making and offline learning allows the agents to balance the real-time responsiveness and learning capability. This strategy not only enables the agents to make timely decisions in dynamic environments but also continuously enhances their decision-making abilities through offline learning, thereby achieving a more intelligent and adaptive hierarchical admission control system.

## 5. Simulation Analysis

### 5.1. Simulation Parameter Settings

According to the relevant parameters of the Iridium constellation, a simulation environment is built by STK to obtain the satellite coverage time of each ground zone. Then, we establish a simulation system, and implement an intelligent hierarchical admission control strategy through MATLAB. Its simulation parameters are shown in Table 4.

### 5.2. Simulation Result Analysis

In the field of DRL, the convergence of algorithms is regarded as a key indicator for evaluating the performance and effectiveness of the algorithm. Especially when applied to LEO satellite communication systems, the convergence speed and stability of the reward function directly reflect the optimization level of the algorithm and its adaptability under different circumstances. Therefore, we observed the changes in the reward function during model training to obtain the optimal settings for model parameters. Furthermore, in simulation experiments, the proposed IHAC strategy is compared with DAQC and WOA in terms of channel user quantity, successful access probability, and drop call rate, demonstrating the applicability and superiority of the proposed strategy.

**Figure 3:**
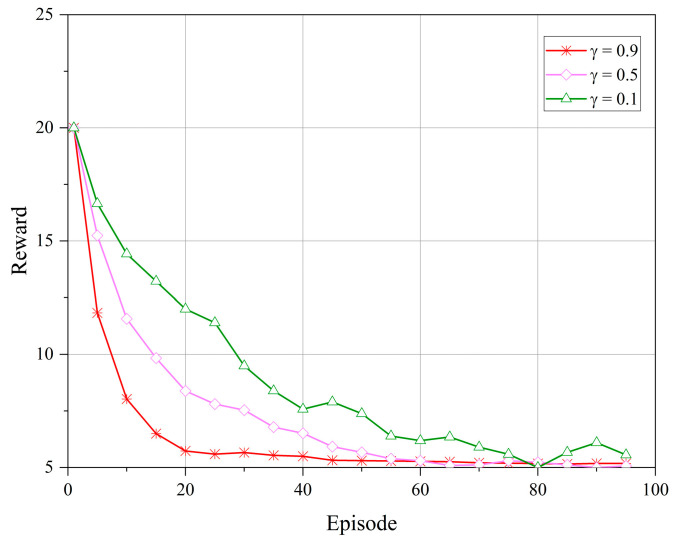
Reward of the upper-level controller under different reward discount factors.

**Figure 4:**
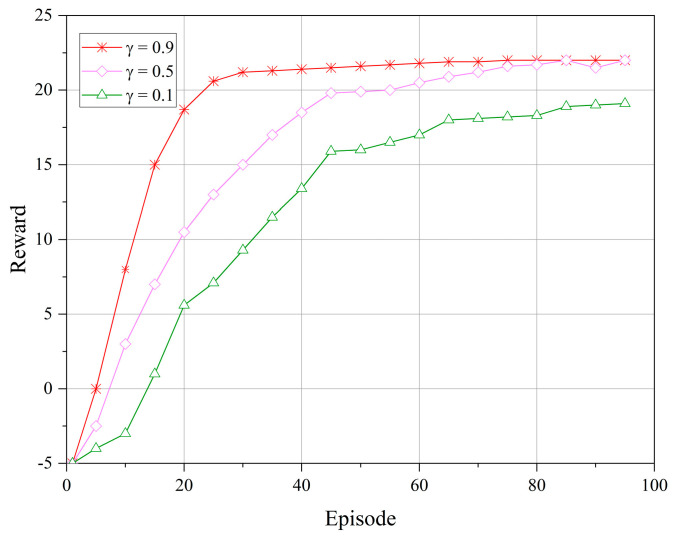
Reward of the lower-level controller under different reward discount factors.

Figure 3 and Figure 4 depict the reward performance of the upper- and lower-level controllers during the training process, where the *x*-axis represents the number of training episodes and the *y*-axis represents the reward value. In DRL algorithms, the objective is to learn to select the optimal policy to maximize the cumulative reward. The reward discount factor can be used to balance the immediate reward at the current time step and the future rewards, with a larger discount factor focusing more on future rewards, allowing the controller to consider the long-term impact of actions and promoting algorithm convergence. Therefore, in our experiments, we set the reward discount factors as γ={0.9,0.5,0.1}. From the figures, it can be observed that the reward values tend to stabilize during the algorithm iterations. However, when γ=0.9, the reward function stabilizes more rapidly and remains stable without significant fluctuations over a longer period. This indicates that when γ=0.9, the upper- and lower-level controllers exhibit a higher adaptability to different communication scenarios and service demands in the LEO satellite communication system.

**Figure 5:**
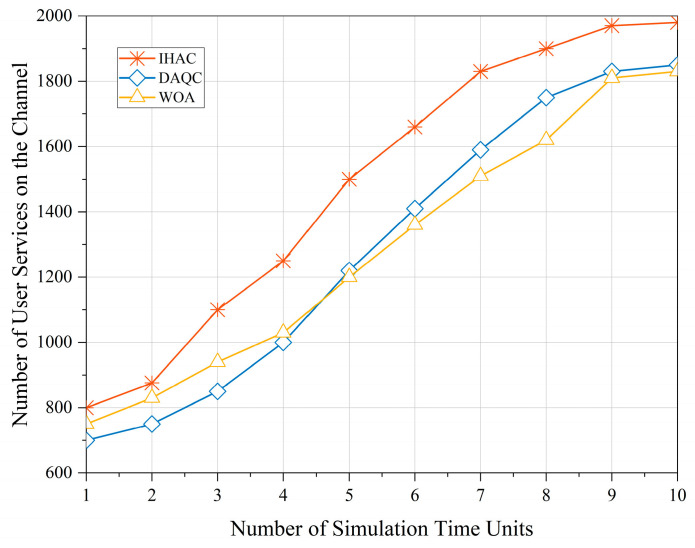
Number of user services on the channel.

In this paper, simulation verification is conducted using a time division approach, where a single resource allocation time window is divided into 10 units of simulated time. Figure 5 presents the changing trend of the number of user services on the channel as the simulation progresses. From the figure, it is evident that the intelligent hierarchical admission control strategy interacts continuously with beam resources and user service states. This interaction, constrained by the reward function, maximizes the utilization of beam resources. By continuously interacting with beam resources and user service states through hierarchical admission control, the maximum utilization of beam resources is achieved, thereby avoiding resource idleness.

The proposed strategy in this paper shows a significant improvement in channel utilization after sufficient training. It not only avoids resource wastage but also effectively meets the access demands of user services. Compared to DAQC and WOA, the proposed strategy achieves the highest received service count of 280 and 320, respectively, while the accepted service count is increased by 22.95% and 21.19%, respectively. The results highlight that the proposed intelligent strategy, after training, significantly enhances the utilization of channels, not only preventing resource wastage but also efficiently satisfying the access requirements of user services.

**Figure 6:**
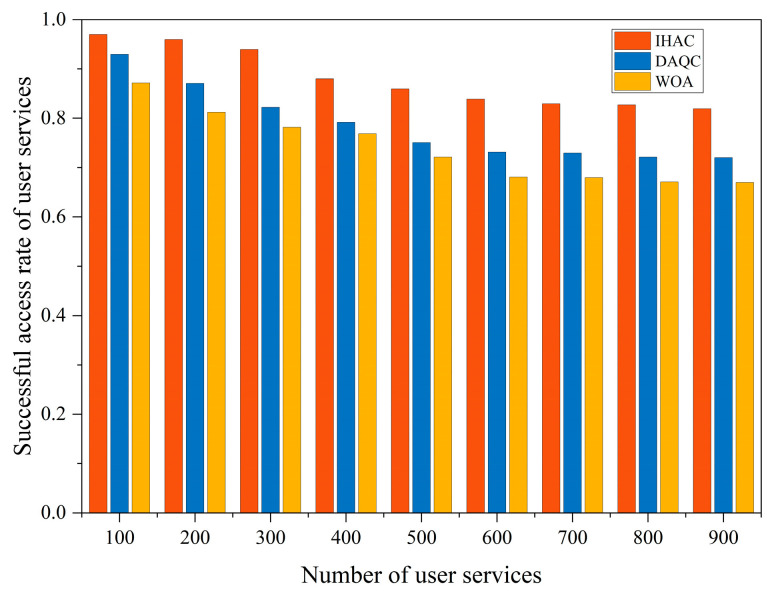
Rate of successful service access.

The rate of successful service access is a critical parameter that reflects user service satisfaction and is one of the core goals of satellite networks. A high rate of successful service access implies that services are more likely to be effectively provided. Especially in scenarios with numerous service demands, terminal users need to compete for limited beam resources. Therefore, the level of this indicator significantly impacts user experience. As shown in Figure 6, as the number of services increases, the rate of successful service access gradually decreases. This is due to network congestion that might prevent accommodating all service access requests. However, the strategy proposed in this paper maintains a relatively high rate of successful service access across various service quantity scenarios. Compared to DAQC and WOA, the average success access rate is increased by 12.12% and 19.05%, respectively. This demonstrates the strategy’s remarkable adaptability in highly competitive environments.

**Figure 7:**
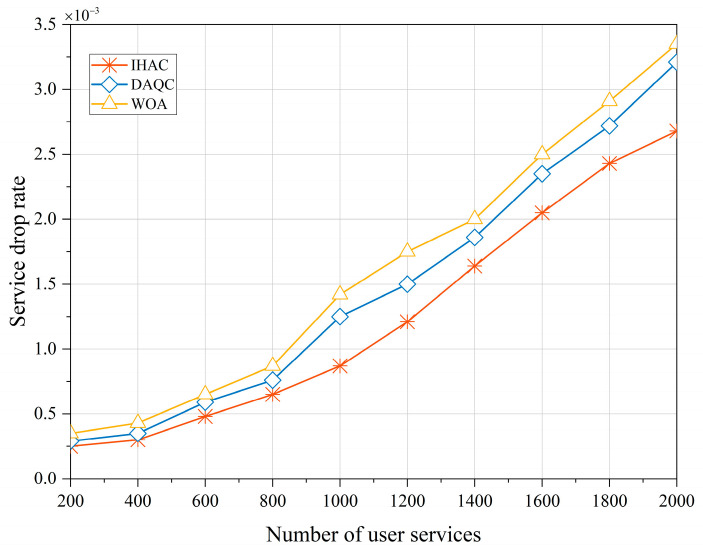
Drop call rate of user services.

Compared to services that have not been accessed yet, services that are interrupted after accessing are more challenging to accept. This not only means that the resources allocated to the service are released but that this also requires reapplication and resource competition to continue the service. As shown in Figure 7, the service drop rate changes based on different service quantities. The graph clearly indicates that the proposed strategy maintains a lower drop rate across various service quantity scenarios. This further underscores the strategy’s efficiency in resource allocation and admission decisions, reducing service interruptions and dropped calls, thus enhancing user experience and satisfaction.

**Figure 8:**
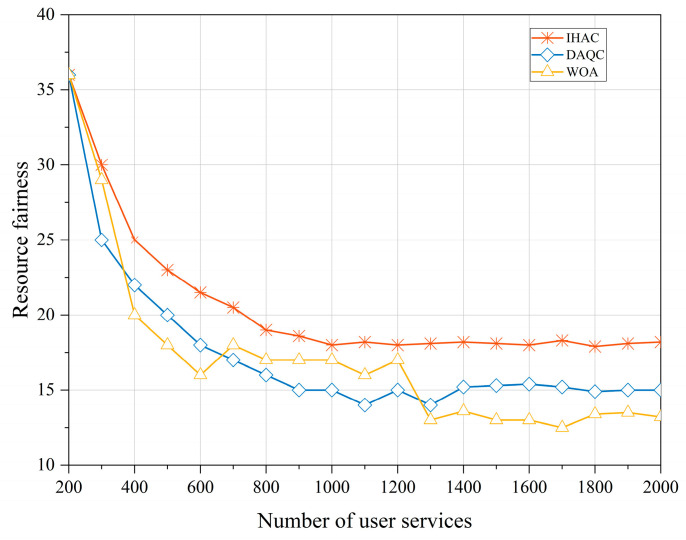
Fairness in resource allocation.

The fairness of resource allocation is defined by the ratio of allocated resources to requested resources, which reflects the satisfaction of ground zone services. Achieving fair resource allocation can reduce unnecessary resource competition. An analysis of Figure 8 indicates that in the initial stages of the strategy, due to the relatively low number of services initially connected to the satellite and the relatively abundant system resources, the fairness of resource allocation is relatively high. However, as the number of requested communication services gradually increases, differences in dynamic priorities among ground zones and within each zone’s services become more apparent, leading to a gradual decrease in the fairness of resource scheduling. During the process where the number of services reaches 1000, the strategy proposed in this paper reaches and maintains stable resource fairness at the fastest pace. It outperforms DAQC and WOA in terms of resource fairness. This demonstrates the strategy’s exceptional adaptability when facing dynamic and diverse priorities. It maintains high fairness even as the number of services increases, showcasing its ability to effectively handle varying priority scenarios.

Finally, we show the performance comparison between IHAC and baseline algorithms with average metric data, as shown in Table 5. In addition, we analyze the time and space complexity of IHAC. As we mentioned before, the ground zone and user service status characteristics in each zone are reported to the upper and lower controllers, respectively, in order to train and run the DDPG and DQN in the controller. Since all training and running tasks are performed by the controller, most of the computation and storage cost is incurred in the controller.

In the controller, the computation related to DRL consumes most of the resources, and we focus on analyzing its time and space complexity. During the inference and operation of DDPG and DQN, their computational overhead mainly depends on the number of nodes in the input layer. Specifically, if M represents the number of ground zones, K represents the characteristic dimension of each ground zone, N represents all user services within the satellite coverage, and L represents the characteristic dimension of user services, then the input layer of DDPG consists of MK units, and the input layer of DQN consists of NL units. Then the time complexity can be expressed as $O(|MK{|}^{2})+O(|NL{|}^{2})$. Since DAQC also uses the Markov decision process to model the problem, which is similar to the DRL modeling process, we estimate that its computational complexity is comparable to IHAC. However, for swarm intelligence heuristic algorithms such as WOA, the computational complexity is related to the dimension of search space *D* and the time complexity *f* of the fitness function. That is, it can be roughly expressed as $O(|M{|}^{2}(D+f))+O(|N{|}^{2}(D+f))$. In addition, the space complexity of the upper and lower controllers can be expressed as O(MK)+O(NL). However, in the training process, we only need to use a set of samples to carry out the forward propagation of DDPG and DQN. So, the actual space complexity is much less than O(MK)+O(NL).

We can find that DL techniques require more computation and storage costs compared to traditional strategies. However, the IHAC we consider can not only achieve the adaptive ability to the dynamic environment through the feature extraction of massive data, but also achieve the dual goals of efficient utilization of beam resources and service fairness.

## 6. Conclusions

This paper proposes an intelligent hierarchical admission control (IHAC) strategy based on DRL for LEO satellites. The strategy addresses the resource allocation and admission control challenges in the field of multi-beam satellite communications, specifically focusing on intelligent admission control for ground zone services within a multi-beam coverage area. The strategy employs DDPG and DQN algorithms to create a hierarchical control strategy that coordinates global resource scheduling with local service admission. The integration of user service models and adaptive dynamic priority models ensures a balance between resource utilization and fairness.

By introducing well-designed state feature inputs and reward mechanisms, the strategy guides decision-making to achieve efficient resource utilization and fairness. In summary, the IHAC strategy proposed in this paper fills the gap in the field of satellite multi-beam communications by providing a comprehensive and effective hierarchical admission control strategy. Leveraging the power of DRL, this strategy can cater to diverse service demands, ensuring both efficient resource utilization and service fairness. It presents a solution for the optimization and advancement of multi-beam satellite communication systems.

## Figures and Tables

**Table 1:** User service model parameters.

Notation	Definition
$B_{total}$	The total bandwidth of the multi-beam satellite
$P_{total}$	The total power of the multi-beam satellite
$U_{m,n}$	The nth user service model in the mth ground zone
$B_{m,n}$	Requested bandwidth of $U_{m,n}$
$P_{m,n}$	Requested power of $U_{m,n}$
$C_{m,n}$	Requested capacity of $U_{m,n}$
$V_{m,n}$	Initial priority of user services $U_{m,n}$
$Ts_{m,n}$	The arrival time of the user’s service $U_{m,n}$
$S_{m,n}$	The time required to complete the $U_{m,n}$
t	The current time
ST, ET	The start and end time of the current resource allocation time window
$DP_{m,n}$	Dynamic priority model for $U_{m,n}$

**Table 2:** DRL formulation parameters.

Notation	Definition
$N_{m}$	The number of user services requests in the mth ground zone
$Pr_{m}$	The total dynamic priority of user services in the mth ground zone
$Wt_{m}$	The total waiting time of user services in the mth ground zone
$B_{m},P_{m},C_{m}$	The total requested bandwidth, power, and capacity of user services in the mth ground zone
${\hat{B}}_{m,n},{\hat{P}}_{m,n},{\hat{C}}_{m,n}$	The ratio of the requested bandwidth, power, and capacity of the nth user service to the total requested resources in the mth ground zone
$SPR_{m}$	The total dynamic priority of successfully admitted services in the mth ground zone
$SPR_{ave}$	The average total dynamic priority of successfully admitted services across all ground zones
*DWT_m_*	The total waiting time of services not admitted in the mth ground zone
$DWT_{ave}$	The average total waiting time of services not admitted across all ground zones
$SWT_{m}$	The total waiting time of successfully admitted services in the mth ground zone
$RP_{m}$	The reward or penalty for the resource utilization rate of the ground area admission control strategy
$RU_{m}$	The ratio of resources allocated to user services within the mth ground zone

**Table 3:** Intelligent hierarchical admission control strategy.

1: Initializing the neural network parameters θ, ω, and μ in the upper and lower-level controllers. Let $\theta^{\prime }=\theta$, $\omega^{\prime }=\omega$, and $\mu^{\prime }=\mu$. 2: Initializing the experience replay buffers, training episode TE in the upper and lower-level controllers. 3: Initializing the sampling episode SE with 0. 4: Initializing the state features $s_{t}^{up}=(s_{t,1}^{up},s_{t,2}^{up},\ldots ,s_{t,m}^{up},\ldots ,s_{t,M}^{up},s_{t,B}^{up},s_{t,P}^{up})$. 5: **For** each sampling episode SE, **do:** 6: The online actor network of the upper-level controller computes the scheduling strategy of beam resources for each ground zone based on the input state $s_{t}^{up}$, i.e., $a_{t}^{up}=\pi (s_{t}^{up}|\theta )$. Subsequently, the scheduling strategy $a_{t,m}^{up}$ is transmitted to the corresponding lower-level controller. 7: The lower-level controllers deployed in each ground zone combine the local service information with the upper-level strategy $a_{t,m}^{up}$ to make decisions. The online DQN then makes admission control decisions for each user service within the ground zone based on the input state, i.e., $a_{t,m}^{down}=\pi (s_{t,m}^{down}|\mu )$. 8: The upper-level beam resource scheduling strategy and the lower-level user service admission control strategy are executed, resulting in states $s_{t}^{up}$ and $s_{t,m}^{down}$, as well as rewards $r_{t}^{up}$ and $r_{t,m}^{down}$. These components form experience samples $\left(s_{t}^{up},a_{t}^{up},s_{t+1}^{up},r_{t}^{up}\right)$ and $\left(s_{t,m}^{down},a_{t,m}^{down},s_{t+1,m}^{down},r_{t,m}^{down}\right)$, which are stored in the upper-level and lower-level experience replay pools, respectively. 9: Let $s_{t}^{up}=s_{t+1}^{up}$ and $s_{t,m}^{down}=s_{t+1,m}^{down}$. 10: If SE>TE: 11: Samples $N(s_{i}^{up},a_{i}^{up},s_{i+1}^{up},r_{i}^{up})$ and $N(s_{i,m}^{down},a_{i,m}^{down},s_{i+1,m}^{down},r_{i,m}^{down})$ are obtained through random sampling, and then the neural networks of the upper-level and lower-level controllers are trained and updated using Equations (15)–(23). 12: **End for.**

**Table 4:** Parameter setting.

Parameter	Value
Satellite total power, $P_{total}$	30 dBW
Satellite total bandwidth, $B_{total}$	500 MHz
Satellite height	600 km
Number of services within satellite coverage area	2000
Number of ground zones	40
Simulation time unit length, Ts	50 ms
Initial priority of user services, $V_{i}$	1, 2, 3, 4, 5
Initial required capacity of services, $C_{i}$	[2, 4, 8, 12, 16, 24] × 300 bps
Discount factor for rewards, γ	0.9
Soft update learning rate, τ	0.001
Training episode, TE	300
Experience replay buffers	5000
Sampling batch size	128

**Table 5:** Performance comparison of IHAC with baseline algorithms.

Metrics	IHAC	DAQC	WOA
Average number of user services on the channel	1486.8	1295	1298
Average call drop rate of user services	1.256	1.488	1.623
Average probability of successful service access	0.881	0.785	0.739
Average fairness in resource allocation	20.668	17.526	17.168

## Data Availability

Not applicable.
